# The Act of Prevention: Knowledge, Attitudes, and Perception Among Caretakers of Kidney Disease Patients in Rural Wardha District of Central India

**DOI:** 10.7759/cureus.23058

**Published:** 2022-03-11

**Authors:** Gaurav Sahu, Sunil Kumar, Sourya Acharya, Dhruv Talwar, Akhilesh Annadatha, Mansi Patel, Twinkle Pawar, Divit Shah, Neha Phate, Prerna Verma

**Affiliations:** 1 Department of Medicine, Jawaharlal Nehru Medical College, Datta Meghe Institute of Medical Sciences (Deemed to be University), Wardha, IND

**Keywords:** internal medicine in rural areas, caregiver, perception, attitude, knowledge, chronic kidney disease

## Abstract

Background

There is a continual rise in the prevalence of non-cancerous conditions such as chronic kidney disease (CKD) owing to an enormous load of diabetes, hypertension, and vascular diseases. A positive attitude and healthy lifestyle for CKD prevention can only be followed when the masses are well aware and educated about the disease. This study aimed to compare, correlate, and evaluate the distribution of knowledge, attitudes, and perceptions among relatives or caretakers of patients with kidney disease or at risk of the disease.

Methodology

This cross-sectional study aimed at obtaining data on the knowledge, attitudes, and perceptions using the Chronic Kidney Diseases Screening Index questionnaire from the relatives of CKD patients. All data were computed and analyzed using SPSS version 28.0 (IBM Corp., Armonk, NY, USA).

Results

The majority of the relatives of CKD patients had poor knowledge (63.6%) and poor attitude (51.6%) levels. On the contrary, most respondents had good practices (52.8%) level toward the risk for CKD. A significant correlation was noted between education and knowledge (p < 0.050). A significant association was also observed between education and occupation with attitude (p < 0.001 and p < 0.050, respectively). Additionally, a significant association was noted between age and perception (p < 0.001).

Conclusions

Informed and well-educated populations are less prone to acquire or progress to CKD. From this study, we can understand the need for improvement in public knowledge, which has the potential to help in saving the lives of many patients progressing toward end-stage renal diseases.

## Introduction

According to the National Kidney Foundation Kidney Diseases Outcome Quality Initiative, chronic kidney disease (CKD) is summarized as (a) glomerular filtration rate (GFR) of less than 60 mL/minute/1.73 m^2^ for three months or more, with or without kidney damage. (b) Kidney injury for more than three months, as indicated by anatomical or functional abnormalities in the kidney, with or without fall in GFR, that manifest as signs of renal diseases, such as (i) alterations in the composition of blood (abnormal erythrocyte sedimentation rate) or urine (albuminuria > 30 mg/24 hours; urine albumin-creatinine ratio > 30 mg/g), (ii) abnormalities detected through histology, and (ii) structural abnormalities detected through imaging [1].

Approximately 100,000 new cases of kidney disease, especially in the end stage, are reported annually in India due to late referral from primary healthcare and lack of awareness among the population as well as general practitioners [2]. Patients with end-stage renal disease (ESRD) are merely the forerunners of a massive wave of patients in the early stages of CKD who require more care to slow the course of the disease [3]. Early detection is the only mainstay for delaying progression. One such important modifiable risk factor is elevated blood pressure regardless of any previous renal injuries [4]. Literature has enough evidence to show that renal damage and progression to renal failure are slowed by reducing blood pressure. Diabetes-related renal disease is increasing at a rate of 10% annually, and if this trend continues, the number of new patients diagnosed with diabetic renal failure will more than double in the next eight years [5]. The prevalence of ESRD among smokers is higher and estimated to be as much as 5.9 times more among heavy smokers (>15 pack-years) [6].

To estimate the level of awareness among the population and their attitude toward the same, the Knowledge-Attitude-Perception (KAP) model was used [7]. This model has long been used by healthcare providers and management [8,9]. In the early 1980s, Schwartz was the original introducer of this concept [10]. The key idea of this concept was that people’s attitudes and behaviors would change once they understood the fundamentals of changing their health state. This KAP transition is not achievable in the short term and needs patient and healthcare therapeutic relationships over time. Knowing the precise awareness and knowledge level of the population will not only help improve the health status but can also be used to assess illness states and alter intervention methods if required.

Although the prevalence and incidence of kidney disease are high, studies examining its actual knowledge level, both locally and nationally, are limited. A literature search on PubMed, Medlar, and Google did not show any published data from India. The purpose of this research is to describe the participants’ knowledge, attitudes, and perceptions among caretakers of patients with kidney disease admitted to our rural hospital.

## Materials and methods

This prospective observational study was conducted in Acharya Vinoba Bhave Rural Hospital, a tertiary-care rural teaching hospital at Wardha district located in central India, at the Department of Medicine, which has a well-equipped dialysis center with 24-hour hemodialysis and peritoneal dialysis facilities. The study was conducted after obtaining approval from the Institutional Ethical Committee of Datta Meghe Institute of Medical Sciences (approval number: 2020-21/8864). Written and verbal informed consent was taken from all individuals participating in the study. The duration of the study was from September to October 2021 under undergraduate MBBS Short Term Studentship (STS) research work approved by the Indian Council of Medical Research (STS 04180-2020).

Data collection tool and validation of the questionnaire

Relatives accompanying patients with CKD who were admitted for hemodialysis were enrolled in this study. Face validity was determined by giving the questionnaire to 10 individuals (five randomly selected females and males) who fulfilled the inclusion criteria to see if it was coherent, clear, simplistic, easy, and intelligible. These individuals were excluded from the final analysis and were not further categorized based on their demographic variables other than gender. Three experts (a research supervisor, a co-supervisor, and a medical registrar) were given the questionnaire to assess if the information fulfilled the study’s goals for content validity. Thus, no reliability test was performed because this questionnaire was based on two surveys that had very high-reliability scores (Cronbach’s alphas for knowledge, attitude, and perception were 0.87, 0.73, and 0.78, respectively) [11].

Section 1 of the questionnaire comprised questions about the participants’ demographic parameters as well as questions about the KAP of relatives of CKD patients, followed by section 2 which included a set of 25 items divided into three broad domains, namely, knowledge, attitudes, and perceptions.

The knowledge domain comprised 10 questions to measure the basic knowledge and understanding of renal diseases leading to their diagnosis and treatment. Each question was answered on a four-point grading scale (“Yes,” “No,” “Do Not Know,” and “Unsure”).

The attitudes section included eight items that recorded relative’s attitudes toward their ability to recognize symptoms, attitude toward self-awareness, social awareness, emotional intelligence, psychometric, and critical reasoning behavior in knowing that they are relative of CKD patients. A two-point grading scale (“Yes” or “No”) was used to test eight items in the attitude domain. A good attitude was scored 1, and a poor attitude was scored 0. Finally, to test the various hypothetical practices associated with a diagnosis of kidney disease, the perception domain was created. It comprised seven items with each item measured on a four-point Likert-based scale (“Very Unlikely,” “Unlikely,” “Likely,” and “Very Likely”). The good perception was at the higher end of the scale. Scores of more than 70% were defined as high knowledge, good attitude, and good perception [12].

Disease definitions

After the KAP questionnaire had been filled out, participants were given free screening for kidney disease, diabetes, and hypertension. The Modification of Diet in Renal Disease (MDRD) definition of chronic renal disorder is the presence of albumin in the urine (>30 mg/dL on repeat assessment) and/or a GFR of 60 mL/minute/1.73 m^2^ regardless of race [13].

According to the updated guidelines, hypertension was defined as a single sitting reading of greater than 160/100 mmHg, or repeated average measurement of greater than 140/90 mmHg, or already on prescribed anti-hypertensive medications.

Diabetes was defined as a fasting plasma glucose level of 126 mg% and two-hour plasma glucose levels after the oral glucose tolerance test of 200 mg%, or the ongoing use of anti-hyperglycemic medications.

Participants with poor diabetes control, hypertension, or lacking awareness of diabetes and hypertension were considered as high risk for CKD and included in the study.

Statistical analysis

The SPSS version 28.0 (IBM Corp., Armonk, NY USA) software was used for all data analyses. Continuous variables were presented as means and standard deviation, and categorical variables were summarized as frequency and percentages. Fisher’s exact test was used to determine differences in unweighted proportions between groups, and the chi-square test was used for determining independence. P-values of <0.05 were considered significant.

## Results

Out of the 250 participants, 137 (54.8%) were males and 113 (45.2%) were females. Respondents’ age was categorized into three groups, namely, 18-40, 41-60, and >60 years. Most participants were young adults, that is, 18-40 years old (50.4%), and the majority were females (27.6%). The mean age was 54.6 ± 12.5 years (range = 18-90). There was a broad array of levels of education, with 45.6% having a college education or being a college graduate, 21.6% with secondary school education, 16.8% with primary school education, and 16% were illiterate. The majority of the participants were unemployed 158 (63.2%). Of those who were employed (92, 36.8%), 23.2% were males and 13.6% were females. The demographic data of the participants are shown in Table 1.

**Table 1:** Respondents’ sociodemographic characteristics (n = 250).

Variables	Male, Number (%)	Female, Number (%)	Total, Number (%)
Sex	137 (54.8)	113 (45.2)	250 (100.0)
Age (years)			
18–40	57 (22.8)	69 (27.6)	126 (50.4)
41–60	47 (18.8)	23 (9.2)	70 (28.0)
>60	33 (13.2)	21 (8.4)	54 (21.6)
Education			
Never	22 (8.8)	18 (7.2)	40 (16.0)
Primary school	27 (10.8)	15 (6.0)	42 (16.8)
Secondary school	34 (13.6)	20 (8.0)	54 (21.6)
College/Graduate	54 (21.6)	60 (24.0)	114 (45.6)
Occupation			
Employed	58 (23.2)	34 (13.6)	92 (36.8)
Unemployed	79 (31.6)	79 (31.6)	158 (63.2)

Knowledge, attitudes, and practices behaviors

Knowledge

Participants’ mean scores on the knowledge scale were 5.44 (SD = 2.15; range = 0-9), and 91 individuals out of 250 (36.4%) had good knowledge. However, no individuals correctly answered the entire 10 items of the knowledge section, and 177 (70%) participants achieved more than 50% of the correct scores. Evaluation of the inputs on the knowledge scale by the relatives of CKD patients is shown in Table 2.

**Table 2:** Responses to knowledge scale in the Chronic Kidney Disease screening index.

Questions	Yes, Number (%)	No, Number (%)	Don’t know, Number (%)	Unsure, Number (%)
Do you think high blood pressure can cause kidney disease?	164 (65.6)	32 (12.8)	48 (19.2)	6 (2.4)
Do you think that high blood sugar (diabetes mellitus) can cause kidney disease?	169 (67.6)	34 (13.6)	42 (16.8)	5 (2.0)
Can drinking alcohol cause kidney disease?	173 (69.2)	27 (10.8)	38 (15.2)	12 (4.8)
Can a person tell if he/she has kidney disease just by the color, quality, or smell of his/her urine?	132 (52.8)	46 (18.4)	56 (22.4)	16 (6.4)
Kidney disease can only be diagnosed by a test at the hospital?	168 (67.2)	37 (14.8)	33 (13.2)	12 (4.8)
Kidney disease can be prevented if you follow the advice of a medical doctor?	195 (78.0)	20 (8.0)	26 (10.4)	9 (3.6)
Do the kidneys control body temperature?	127 (50.8)	34 (13.6)	83 (33.2)	6 (2.4)
Do the kidneys filter waste products from the blood?	214 (85.6)	10 (4.0)	16 (6.4)	10 (4.0)
Is dialysis a form of treatment for kidney disease?	166 (66.4)	58 (23.2)	19 (7.6)	7 (2.8)
Antibiotics are a type of medicine that are used to treat certain infections such as tuberculosis and urinary tract infections, among other things. Antibiotics are a form of treatment for kidney disease?	127 (50.8)	54 (21.6)	55 (22.0)	14 (5.6)

Attitude

The mean score on the attitude scale was 5.79 (SD = 1.48; range = 1-8). In total, 20 individuals scored the maximum indicating an exceptionally good attitude. Further, 48.4% scored more than 70%, suggesting a good attitude. And 84.8% were curious and had the idea of learning about kidney problems. In total, 80.8% were worried about their chances of survival if they found out that they have kidney problems. And 81.2% thought that the cost of kidney disease is a problem for them. Evaluation of the inputs on the attitude scale by the relatives of CKD patients is shown in Table 3.

**Table 3:** Responses to attitude scale in the Chronic Kidney Disease screening index.

Questions	Yes, Number (%)	No, Number (%)
Have you thought that you may have kidney problems?	79 (31.6)	171 (68.4)
Do you like the idea of learning all that you can about kidney problems?	212 (84.8)	38 (15.2)
If you found out that you have kidney problems, would you be worried about your future?	193 (77.2)	57 (22.8)
Would you be worried about your reputation in the community if you found out that you have kidney disease?	110 (44.0)	140 (56.0)
Would you be worried about your ability to work if you found out that you have kidney problems?	192 (76.8)	58 (23.2)
Would you be worried about your chances of survival if you found out that you have kidney problems?	202 (80.8)	48 (19.2)
Do you think that kidney disease is a problem in India?	135 (54.0)	115 (46.0)
Do you think that the cost of kidney disease would be a problem for you?	203 (81.2)	47 (18.8)

Perception

The percentages of responses by study participants to questions related to practice among relatives of patients with kidney disease are summarized in Table 4. The mean score on the practices scale of the CKD Screening Index was 20.94 (SD = 4.04; range = 9 to 28). Of the 132 participants, 52.8% had good perception levels. Further, 48.4% wanted to seek care from a traditional healer, 86.4% preferred treatment at the hospital, and 90% were willing to see a medical doctor if they found out that they have kidney problems.

**Table 4:** Responses to the perception scale in the Chronic Kidney Disease screening index.

Questions	Very unlikely, Number (%)	Unlikely, Number (%)	Likely, Number (%)	Very likely, Number (%)
How likely would you be to seek care from a traditional healer?	75 (30.0)	54 (21.6)	68 (27.2)	53 (21.2)
How likely would you be to seek self-treatment at home?	78 (31.2)	72 (28.8)	58 (23.2)	42 (16.8)
How likely would you be to seek care at a hospital or health clinic?	4 (1.6)	30 (12.0)	47 (18.8)	169 (67.6)
Would you be willing to be contacted by cell phone regarding the care of your kidneys?	22 (8.8)	37 (14.8)	71 (28.4)	120 (48.0)
Would you be willing to be contacted by email regarding the care of your kidneys?	34 (13.6)	33 (13.2)	71 (28.4)	112 (44.8)
Herbal or natural medications are commonly used to treat health problems. Herbal or natural medications may include herbs, teas, foods, creams, lotions, potions, and soups that are used to treat health problems. How likely would you be to use herbal or natural medications if you found out that you have kidney disease?	38 (15.2)	59 (23.6)	98 (39.2)	55 (22.0)
How likely would you be willing to see a medical doctor if you found out that you have kidney problems?	3 (1.2)	22 (8.8)	60 (24.0)	165 (66.0)

**Figure 1:**
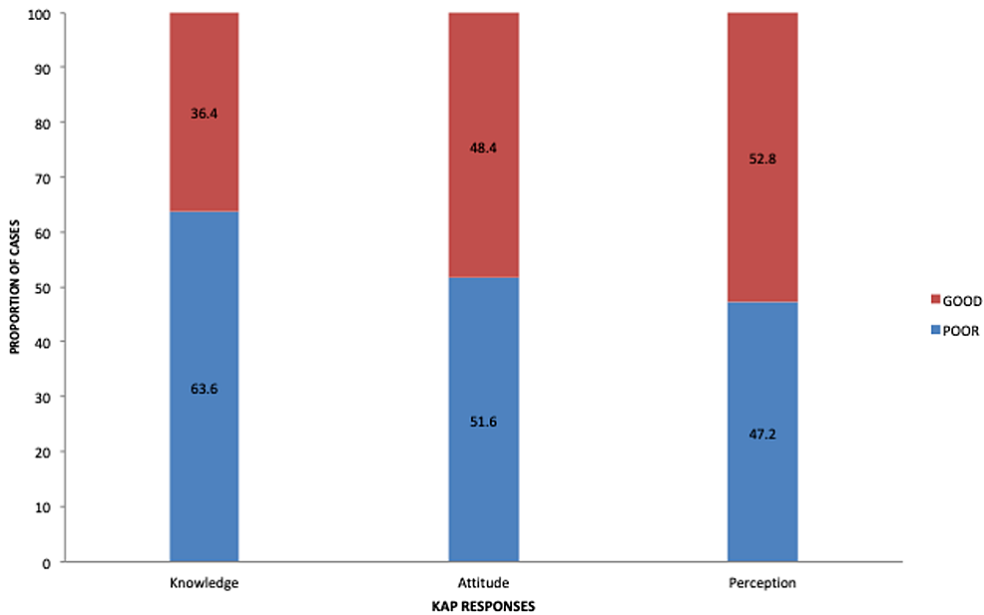
Respondents’ knowledge, attitude, and practices toward the risk for chronic kidney disease. KAP: knowledge, attitude, and practices

Descriptive analysis of the levels of KAP domains of the relatives toward the risk for CKD is shown in Figure 1. The majority of respondents in this study had poor knowledge levels (63.6%) and poor attitude levels (51.6%). In contrast, most respondents had good practices levels (52.8%) toward the risk for CKD.

The study showed a significant association between education and knowledge (p = 0.025) toward the risk of CKD. Participants who were in school and were college graduates had better knowledge than illiterate participants. There was no significant association between occupation and knowledge (p = 0.133) toward the risk of CKD. A significant association between education and attitude (p = 0.001) toward the risk of CKD was observed as well as between occupation and attitude (p = 0.050). There was a significant association of age with the perception (p ≤ 0.001) of the risk of CKD where younger people had a better perception than older ones. No significant association between education and the perception (p = 0.324) of the risk of CKD was observed. Table 5 shows additional associations between sociodemographic variables and relatives’ knowledge, attitudes, and practices regarding the risk of CKD.

**Table 5:** Association between sociodemographic variables and knowledge, attitudes, and practices of the relatives toward the risk of chronic kidney disease (n = 250). *: significant p-values.

	Knowledge				Attitude				Perception			
	Poor, Number (%)	Good, Number (%)	χ^2 ^(Df)	P-value	Poor, Number (%)	Good, Number (%)	χ^2 ^(Df)	P-value	Poor, Number (%)	Good, Number (%)	χ^2 ^(Df)	P-value
Sex												
Male	91 (36.4)	46 (18.4)	1	0.307	73 (29.2)	64 (25.6)	1	0.557	69 (27.6)	68 (27.2)	1	0.270
Female	68 (27.2)	45 (18.0)			56 (22.4)	57 (22.8)			49 (19.6)	64 (25.6)		
Age (years)												
18–40	88 (35.2)	38 (15.2)			72 (28.8)	54 (21.6)			49 (19.6)	77 (30.8)		
41–60	39 (15.6)	31 (12.4)	2	0.109	31 (12.4)	39 (15.6)	2	0.191	31 (12.4)	39 (15.6)	2	<0.001*
>60	32 (12.8)	22 (8.8)			26 (10.4)	28 (11.2)			38 (15.2)	16 (6.4)		
Education												
Never	30 (12.0)	10 (4.0)			20 (8.0)	20 (8.0)			24 (9.6)	16 (6.4)		
Primary school	33 (13.2)	9 (3.6)	3	0.025*	22 (8.8)	20 (8.0)	3	0.001*	18 (7.2)	24 (9.6)	3	0.324
Secondary school	31 (12.4)	23 (9.2)			16 (6.4)	38 (15.2)			26 (10.4)	28 (11.2)		
College/Graduate	65 (26.0)	49 (19.6)			71 (28.4)	43 (17.2)			50 (20.0)	64 (25.6)		
Occupation												
Employed	53 (21.2)	39 (15.6)	1	0.133	40 (16.0)	52 (20.8)	1	0.050*	41 (16.4)	51 (20.4)	1	0.524
Unemployed	106 (42.4)	52 (20.8)			89 (35.6)	69 (27.6)			77 (30.8)	81 (32.4)		

## Discussion

This study utilized the Theory of Planned Behavior that has been used in several previous studies as a key concept in which individual intention is predicted to determine the extent to which they will engage in a behavior at a specific time and place. Depending on internal beliefs and knowledge, an individual’s attitude may be positive or negative, which decides the path to CKD prevention and early detection [14]. Therefore, it is critical to emphasize that those who did not have adequate knowledge and positive attitudes did not report good actions for CKD prevention and early diagnosis.

This study has effectively brought to light a surprising finding that the majority of the individuals who are relatives of CKD patients and residing in the Vidarbha region of Maharashtra have poor knowledge (63.6%), which is similar to the findings of previous studies by Mohd Yusoff et al. and Danguilan et al. [15,16].

Globally, no previous studies have assessed the KAP of the relatives of CKD patients, except a few studies that tried to correlate similar parameters with the general population of an area. One such study was conducted by Oluyombo et al. in a rural setting in southwest Nigeria, where the population was evaluated for their awareness, knowledge, and perception of chronic renal disease. It was surprising to note that of the 454 residents of that region, only a small fraction of approximately 33.7% were acquainted with kidney disease, with 59.3% getting the information from the media and 35.3% from health workers, pointing toward the need for mass public awareness campaigns and promotions. These results are similar to our findings, where 36.4% of the participants were acquainted with kidney disease. There were still many notions regarding the cure of CKD by spiritual means (45.9%) and herbal solutions (47.8%), which was reflected in our survey as well (61.2% would have liked to use herbal medicine for the cure of CKD) [17].

We found that the educational level was highly associated with knowledge and unhealthy attitudes. These findings are comparable with those of other studies reporting that educational level was significantly associated (p < 0.05) with knowledge, indicating that people who had low educational levels had poor knowledge about kidney diseases and poor attitudes toward disease prevention and progression [18,19].

Stanifer et al. found that awareness of the causation, manifestation, and therapeutics of kidney diseases was inadequate in their study [20], which was comparable to the findings of our study. Wright et al. revealed a moderate level of correlation between perceived and objective knowledge about CKD in those who also had low levels of perception about the disease. Very few participants were aware of the etiological causes that can cause or aggravate renal diseases, including diabetes and hypertension. Even those who had these chronic conditions did not have the knowledge that they could end up having renal pathology [21]. However, more than 50% of our study population were aware of the comorbid conditions that can increase the risk of renal disease.

It is now a known fact and statistically well documented by Danguilan et al. that comprehensive and accurate information about the disease can dramatically help individuals improve their health-seeking behavior [16]; on the other hand, insufficient knowledge can lead to dismal health outcomes [22].

This study did not find any correlation between age and knowledge, as reported by Mohd Yusoff et al. [15]. In contrast, Danguilan et al. found that with increasing age there was a lack of knowledge about the disease, suggesting that with increasing age there is a poor cognitive ability causing poor health care [16]. Furthermore, we found no link between gender and knowledge. However, because the relationship varies depending on the geographical location, it is crucial to assess the educational level of people in various parts of the world.

When it comes to the acquisition and maintenance of specific behavioral patterns, one’s attitude toward disease is crucial because it indicates one’s action in response to a diseased state, receptivity to treatment, and upliftment of self-esteem [23]. Therefore, it was essential to evaluate and study the correlation of attitude with age, gender, educational level, and employment. We found a significant association between education and the attitude (p = 0.001) toward the risk of CKD, indicating that individuals who had been in school and college had a good attitude and a better outlook toward the disease than those who never had an education. Moreover, there was a significant association between occupation and attitude (p = 0.050) toward the risk of CKD. This indicates that individuals who were employed had a positive attitude toward accepting the treatment modalities and were more likely to overcome the extra expenditure associated with the morbid condition of the patient, which involves the cost of frequent transportation to the health center, healthy food, and medical supplies. These findings are consistent with a previous study by Bapat et al. [24].

All these studies point toward the lack of awareness and knowledge of CKD and the prevailing misconceptions regarding the disease. Thus, it is the moral duty of the government, medical professionals, and society to minimize the achievable gap and promote educational programs that will help diagnose CKD early and prevent further complications. Constant efforts are required to spread awareness and educate the masses on chronic renal disease and its prevention.

While studies have been carried out in rural central India based on the features and implications of CKD [25-27], this study provides essential data regarding KAP among caretakers of kidney disease patients which has not been studied in detail before, providing important insights for healthcare providers.

Limitations

The main limitations of this study are the small sample size and its single-center study design. Further studies with more participants and multicenter recruitment are required to generalize the findings of this study. Moreover, because this study was carried out in a rural center of central India, the results might differ from studies conducted in urban settings in the future.

## Conclusions

In India, a large number of new cases of kidney disease, especially in the end-stage, are reported annually due to late referral from primary healthcare centers and a lack of awareness among the population as well as general practitioners. Through this research, the knowledge and perception of kidney disease may be improved, at least among the general rural population, leading to reduced morbidity and mortality.
